# Sex education in Poland – a cross-sectional study evaluating over twenty thousand polish women’s knowledge of reproductive health issues and contraceptive methods

**DOI:** 10.1186/s12889-019-7046-0

**Published:** 2019-06-03

**Authors:** Damian Warzecha, Iwona Szymusik, Bronislawa Pietrzak, Katarzyna Kosinska-Kaczynska, Janusz Sierdzinski, Nicole Sochacki-Wojcicka, Miroslaw Wielgos

**Affiliations:** 1 1st Department of Obstetrics and Gynecology, Medical University of Warsaw, Pl. Starynkiewicza 1/3, 02-015 Warsaw, Poland; 2 Department of Medical Informatics and Telemedicine, Medical University of Warsaw, Warsaw, Poland

**Keywords:** ‘Sex education’, ‘Menstrual cycle’, ‘Contraception’, ‘cervical cancer prevention’, ‘Infertility’

## Abstract

**Background:**

Reproductive health is a part of a comprehensive definition of complete physical, mental and social well-being. Sex education is an important aspect of public health. Ignorance, due to the lack of sex education leads to risky sexual behaviors.

Methods: Our cross-sectional study was aimed at investigating a representative group of Polish women’s knowledge about the physiology of the menstrual cycle, contraceptive methods, infertility and cervical cancer prevention. The data were collected by face-to-face interviews and an anonymous electronic questionnaire.

**Results:**

The study group involved 20,002 respondents. Most of the women were of reproductive age (mean 27.7), parous (60.8%), of higher education (71%) and living in large cities (> 500 k citizens, 36.8%). 62.2% of the women gave correct answers to at least 5 of 7 questions concerning the physiology of the menstrual cycle. Three factors had a significant influence on the number of correct answers: higher education (*p* = 0.0001), more frequent gynecological appointments (*p* = 0.0001) and living in a larger city (*p* = 0.002).

Women of higher education level had more often used some form of contraceptive method previously (87% vs. 78.4%, *p* = 0.001), recommended natural family planning methods to their peers (18.4% vs. 15%, *p* = 0.001) and regularly attended gynecological appointments (85.7% vs. 78.8%, *p* = 0.001) when compared with those women with lower educational levels. The three most effective contraceptive methods identified by respondents were: oral contraceptives (71.1% answers), intrauterine devices (50.2%) and parenteral hormonal contraceptives (30.4%). The effectiveness of natural family planning was more often emphasized by women who had never used any contraceptives before (20.1% vs 6.7%). Most of the participants (80.8%) believed that in-vitro fertilization is an effective infertility treatment and should be reimbursed in Poland. Also, 95.2% of the respondents reported that they had undergone a Papanicolaou test within the past 3 years, but only 3% of these women were aware of all the risk factors for cervical cancer mentioned in our survey.

**Conclusions:**

It is very important to improve comprehensive reproductive health education in Poland, especially among women of lower educational levels and living in small centers. In future, educational programs and gynecologists should focus on implementing and improving these aforementioned issues.

**Electronic supplementary material:**

The online version of this article (10.1186/s12889-019-7046-0) contains supplementary material, which is available to authorized users.

## Background

According to the World Health Organization (WHO), reproductive health is a part of a comprehensive definition of complete physical, mental and social well-being. Reproductive health implies that people can have a responsible, satisfying and safe sexual life and they have the capability to reproduce [1]. Each couple has the right to conscious parenthood. Therefore, it is essential to provide people with wide access to contraceptive counseling, as well as familiarity with infertility problems and the possible treatment options. One of WHO’s goals for a global strategy on improving reproductive health includes the implementation of high-quality services for family planning, as well as improved cervical cancer prevention [2].

Sex education is defined as instruction in the physiological and psychological aspects of human reproduction. Ignorance due to a lack of sexual education leads to risky sexual behaviors. Sexual behavior varies from one society to another depending, among other things, on race, ethnicity, gender and socioeconomic status. The mean age of sexual initiation varies from 14 to 16 years of age across most countries [3, 4]. In high socio-economic countries, more than 50% of teenagers between the of ages 15 and 18 have already begun their sexual life [5]. Unfortunately, there is no comprehensive sex education program in Poland for this age group. Even when such classes are available at schools, they are often provided by teachers who are insufficiently trained.

In addition to the consequences of unplanned pregnancy, unsafe sex (more common among adolescents) increases the risk of sexually transmitted diseases (STD), as well as of cervical cancer in women in the future [6]. According to the published data, the rates of protected sex at the time of a person’s first sexual intercourse are very low, at only 38% [7]. The availability, conscious selection and early initiation of contraceptives and safe-sex practices are crucial for the future reproductive health.

In recent decades, there has been a significant decrease in the number of unplanned pregnancies and a reduction in birth rates among adolescents worldwide (e.g., in the United States, the adolescent birthrate has declined from 68 per 1000 in 1970 to 34 per 1000 in 2010; however, in some other high socio-economic countries the rate has decreased by more than half) [8–10]. Nevertheless, it remains that this age group still has an increased risk of unplanned pregnancies and STDs [11].

An additional factor in maintaining reproductive health is an awareness of problems of infertility and the therapeutic management options that are available [12].

Our cross-sectional study was aimed at investigating women’s knowledge of the physiology of the menstrual cycle, contraceptive methods, infertility and cervical cancer prevention. An additional objective was to assess whether levels of knowledge correlate with more effective contraceptive methods being used, opinions about infertility treatment and having regular gynecological appointments and cervical cancer screenings.

## Methods

Our main research phase was preceded by a pilot study carried out at the 1st Department of Obstetrics and Gynaecology, of the Medical University of Warsaw. Women who were admitted to the clinic for various gynecological issues between September and December 2016, were asked to take part in the survey. These data were collected via face-to-face interviews and questionnaire forms with 587 hospitalized women. Afterwards, a similar electronic questionnaire was prepared for a larger and more diverse study group. The study protocol was to make the survey available on the internet for a two-week period. This web-based survey was shared via social media during January 2017 and reached more than 19,000 women respondents. The surveys were written in the Polish language.

The questionnaire used in the study was developed purposely for this study (*an English version of the questionnaire is attached as* Additional file 1). Women were asked to provide anonymous answers to questions regarding selected reproductive health issues that were divided into three sections, as described below. The first section consisted of 7 structured questions that assessed the women’s knowledge about the physiology of menstrual cycles: normal duration of menstrual cycle, menstruation, suspected date and symptoms of ovulation, and site of fertilization. In the second section, multi-choice questions assessed respondents’ knowledge of the effectiveness of the available contraceptive methods: oral contraceptives, parenteral hormonal contraceptives (transdermal patches, subdermal implants or vaginal rings), barrier methods, intrauterine devices, emergency contraception or natural family planning (NFP); as well as their knowledge of the definition of infertility, and the risk factors of cervical cancer and its prevention. Participants were asked to rank the contraceptive methods listed above, thus indicating their knowledge of the most-to-least effective. Their opinions on the availability of infertility treatment services and their reimbursement in Poland was also taken into consideration. The final section comprised the baseline characteristics of the respondents, such as age, parity, education, place of residence, frequency of gynaecological appointments and screening for cervical cancer.

The study was conducted in accordance with the requirements of the Declaration of Helsinki for Medical Research involving Human Subject, and ethical approval was obtained from the Ethics Committee of the Medical University of Warsaw (Reference: AKBE/119/2018).

Statistical analysis was done using the Mann-Whitney U-test for continuous variables and the chi-squared test for categorical variables. Univariate odds ratios (ORs) with 95% confidence intervals were also calculated. A multiple logistic regression model was built to estimate which factors influenced the respondents’ levels of knowledge about reproductive health issues. Statistical Analysis System (SAS) software was used for the statistical analyses. *P*-values below the threshold of 0.05 were considered significant. The preliminary results of the study were previously presented during the 26th European Congress of the European Board and College of Obstetrics and Gynaecology (Paris 2018).

## Results

### Baseline characteristics of the study group

The study group comprised 20,002 respondents (587 in the pilot study and 19,415 in the main study group who responded via the web-based interactive survey). Eighteenrespondents were excluded from analyses due to their incomplete questionnaires. The baseline characteristics of the women in the pilot study and in the internet-based survey were comparable regarding parity, residence and education levels (Table 1). Women who took part in the pilot study were significantly older than those who filled out the web-based form (33.9 vs. 27.5 years old (y.o.), *p* < 0.0001). The pilot study and internet survey results were further evaluated together. Most of the respondents were of reproductive age (mean 27.7, SD 5.2), parous (60.8%), of higher educational level (71%) and lived in large cities (> 500 k (k = thousand) residents, 36.8%).

**Table 1:** Baseline characteristics of the total study group

		Pilot study	Web-based survey	Total study group
		*N* = 587	*N* = 19,415	*N* = 20,002
		Mean +/− SD	Mean +/− SD	Mean +/− SD
Age (years)		33.9 +/−11	27.5 +/− 4.8	27.7 +/− 5.2
Nulliparous		33.9% (199)	39.2% (7614)	39.2%
Parity	1	30.1% (177)	36.2% (7030)	36.2%
	2	19.4% (114)	17.6% (3412)	17.6%
	3	11.0% (64)	5.0% (979)	5.0%
	> 3	5.6% (33)	2.0% (380)	2.0%
Education level	basic	2.2% (13)	0.7% (137)	0.7%
	secondary	32.2% (189)	25.7% (4991)	25.7%
	vocational	9.3% (55)	2.1% (405)	2.1%
	higher	56.2% (330)	71.5% (13882)	71.5%
Place of residence	up to 10 k inhabitants	36.8% (216)	24.2% (4704)	24.2%
	10 k–100 k inhabitants	20.0% (118)	28.5% (5533)	28.5%
	100 k–500 k inhabitants	7.4% (43)	20.6% (3989)	20.6%
	cities > 500 k inhabitants	35.8% (210)	26.7% (5189)	26.7%

*k* thousand

*SD* standard deviation

### Knowledge of the physiology of the menstrual cycle

62.2% of the women answered correctly to at least 5 of 7 questions concerning the physiology of the menstrual cycle (Table 2). The range of correct answers given by respondents varied from 10.4 to 94.4%. The most problematic issue for the respondents was in indicating when the body temperature increases during a normal cycle (10.4% gave correct answers). Women with higher education levels were significantly more likely to answer the questions about reproductive health’s issues correctly (*p* < 0.0001 for each question, Table 3.). Multiple logistic regression analysis was conducted to indicate factors influencing the women’s levels of knowledge regarding reproductive health issues. Among the variable evaluated, it was found that age (adjusted Odds Ratio: aOR 0.98, 95% confidence interval: 95% CI 0.98–0.99), education level (aOR 2.7, 95% CI 1.9–2.9) and infrequent gynaecological appointments (less frequent than every 2 years, aOR 0.74, 95% CI 0.64–0.85) were significant factors influencing whether the woman’s knowledge was sufficient (*p* = 0.0001, *p* = 0.0001, and *p* = 0.0001, respectively).

**Table 2:** The distribution of answers about the menstrual cycle among respondents

Question	Answers (n)			
What is the average length of a menstrual cycle?	20+/− 5 days	24+/− 6 days	28 +/− 7 days	35+/− 10 days
	1.5% (296)	7.1% (1387)	90.1% (17483)	1.3% (249)
Which is the first day of the menstrual cycle?	The last day of menstrual bleeding.	The day when ovulation occurs.	The first day of menstrual bleeding.	It depends on the patient’s choice.
	4.0% (782)	1.1% (214)	94.4% (18328)	0.5% (91)
On which day does ovulation occur?	Immediately after the end of menstruation.	Usually 14 days before the next period.	About the 20th day of the cycle (if it is regular, 25–30 days)	7 days before the expected menstruation.
	2.4% (473)	85.7% (16635)	8.5% (1650)	3.4% (657)
How long does the average period of menstrual bleeding last and what is the average loss of blood?	3–5 days and 30–70 ml	5–7 days and less than 30 ml	7–10 days and less than 30 ml	It doesn’t matter how long the menstruation lasts
	47.5% (9210)	31.0% (6021)	21.0% (4105)	0.41% (79)
In which phase is conception most likely to occur during unprotected intercourse?	During the first (follicular) phase of the cycle	In the ovulation phase	During the second (luteal) phase of the menstrual cycle	During menstrual phase.
	3.2% (619)	83.9% (16287)	12.4% (2403)	0.5% (106)
Which part of the genital tract is the most common site of fertilization?	Vagina	Uterus	Fallopian tube	Ovary
	2.7% (537)	24.5% (4756)	62.5% (12126)	10.3% (1996)
When does the basal body temperature increase during the menstrual cycle?	During menstruation.	At the time of ovulation.	In the second phase of the menstrual cycle (after ovulation).	The cycle phase has no influence on basal body temperature.
	5.3% (1023)	82.6% (16043)	10.4% (2023)	1.7% (326)

**Table 3:** Comparison of answers based on education levels (higher vs others)

Variable	Women with higher education levels vs others	*p* value
Correct answers to at least 5 of 7 questions concerning reproductive health issues	65.9% vs 54.9%	*p* < 0.0001
Prior use of any kind of contraceptive	87% vs 78.4%	*p* = 0.001
Recommendation of natural contraceptive methods to their peers	18.4% vs 15%;	*p* = 0.0001

### Contraceptive methods

84.6% of respondents had used some kind of contraceptive method prior to being surveyed. The three most effective contraceptive methods identified by respondents were: oral contraceptives (71.1% answers), intrauterine devices (50.2%) and parenteral hormonal contraceptives (30.4%). Factors that significantly impacted the selection of these aforementioned contraceptive methods were: parity (aOR 1.17, 95% CI 1.13–1.20, *p* < 0.001), education (aOR 0.83, 95% CI 0.80–0.86, *p* < 0.001), place of residence (aOR 0.9, 95% CI 0.87–0.92, *p* < 0.001) and frequency of gynaecological appointments (aOR 1.27, 95% CI 1.22–1.32 *p* < 0.001). Barrier methods (27.6% of answers) were more often selected by younger women (26.8 y.o. vs. 28 y.o.; *p* < 0.001) and by those of the secondary education level (31% vs. 26.4% of participants from other educational levels; *p* < 0.001).

NFP methods were recommended to friends and acquaintances by 17.4% of respondents, however, only 8.9% of them identified NFP as the most or sufficiently effective contraceptive method. The effectiveness of NFP was more often emphasized by women who had never previously used any other form of contraception (20.1% vs 6.7% in the group of women who had used other forms of contraceptive in the past; *p* < 0.001). The place of residence (10.2% of women from small cities, < 10 k inhabitants vs. 8.5% of citizens of larger cities; *p* = 0.005) had a significant impact on the women’s positive opinion about the efficacy of NFP. Indicating a preference for NFP was independent of the respondent’s age (27.7 y.o. vs. 27.6 in the group of women who thought NFP was ineffective; *p* = 0.1) and independent of the number of correct answers about the physiology of the menstrual cycle (*p*-value for each question > 0.1).

### Infertility

63.9% of respondents knew the definition of infertility. The level of education (aOR 0.90, 95% CI 0.87–0.93), place of residence (aOR 0.96, 95% CI 0.94–0.99) and parity (aOR 1.05, 95% CI 1.02–1.08) were significantly related to the number of correct answers on this matter (*p* < 0.001 for each of three factors mentioned above). 80.8% of participants believed that in-vitro fertilization (IVF) is an effective method and should be reimbursed in Poland. Only 2.9% of them regarded it as ineffective and 2.7% indicated that it should be prohibited. Neither the level of knowledge of reproductive health issues, nor the level of education influenced respondents’ views on IVF (*p* = 0.9).

### Cervical cancer prevention and gynecological appointments

**Fig. 1:**
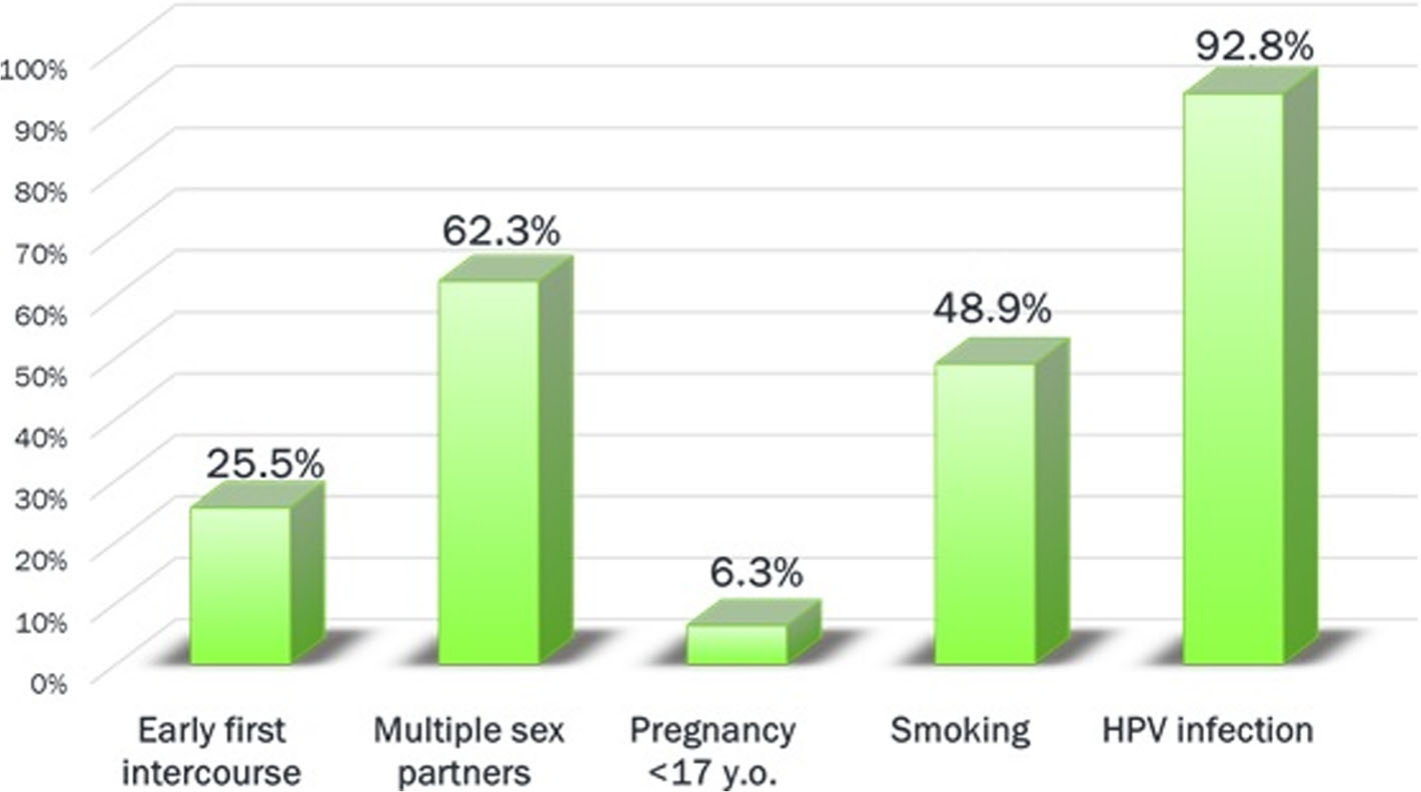
Respondents’ knowledge of cervical cancer risk factors – showing the rates of correct answers

95.2% of the respondents claimed to have undergone a cytological (Pap) smear within the past 3 years. 81.9% of them identified that such screening should be performed at least once a year. Factors such as age (27.6 y.o. vs. 29.2 y.o.; aOR 1.06, 95% CI 1.05–1.07, *p* < 0.001) and the frequency of gynecological appointments (median ‘at least once a year’ vs. ‘every three years’ in the group with irregular attendance; aOR 2.60, 95% CI 2.46–2.83, *p* < 0.001) had a significant impact on respondents’ views about regular attendance at cervical cancer prevention programs (Pap smear performed at least each 3 years). Only 3% of participants were aware of all the risk factors of cervical cancer that were mentioned in the survey. Figure 1. shows the distribution of correct answers concerning cervical cancer risk factors. 83.3% of respondents reported they had attended a gynecologist at least once a year. Women of higher education were more likely to have regular gynecological appointments (at least once a year, 85.7% vs. 78.8% of women of lower education level; *p* = 0.0001).

## Discussion

The purpose of our project was to analyze which female reproductive health issues seem to be the most problematic and require further education. We found no similar studies concerning these problems within a Polish population. Knowledge of physiological changes during the menstrual cycle provides a cornerstone of women’s reproductive health. According to various professionals, parents should be the main source of sex education for youths [13, 14]. This hypothesis is also supported by the results of the large study among British adolescents. For instance, the prevalence of risky sexual behaviors (abandoning contraception during the first intercourse) was higher among teenagers who did not discuss sex matters with their parents [11].

In our study, 62.2% of women had enough knowledge about the physiology of the menstrual cycle (at least 5 of 7 correct answers). It is very important to improve comprehensive reproductive health education in Poland, especially among women at the lower educational levels and living in small centers. Education campaigns that raise awareness about reproductive health should be introduced at all age levels. However, it seems most important to do this among adolescents and young adults who are just entering reproductive age. In most countries, insufficient information about sex matters is given to adolescents because the issues are still considered taboo. Approximately 70% of young people in Britain report that they want access to more information about sex matters from in-school sex education or their parents [15]. According to Kohler et al., teaching about reproductive health issues is not associated with an increased risk of adolescent sexual activity or STDs [16]. Furthermore, adolescents who received comprehensive sexual education had a 50% lower risk of teen pregnancies. A study of 564 teenagers who had already engaged in their first intercourse showed that friends and mass media seemed to be the most popular sources of knowledge about sex behaviors (45 and 41%, respectively). Regrettably, only 15% of adolescents learned from schools and 12.6% from medical professionals [7]. According to Krauss et al., over half of European youths who had started sexual activity did not use any contraceptive methods. Moreover, those who obtained their knowledge of sex from the media more often reported being sexually active. The same study also showed that in Poland, the most trusted source of information about contraception was friends and peers [17]. Sokkary et al., reported that there are especially significant knowledge gaps about available contraceptive methods [18]. Unfortunately, there are no comprehensive sex education programs in Poland. Over 19,000 women took part in the online phase of our survey within the short period of 2 weeks, which proves that there is a great interest in sex education issues in the Polish population.

Higher teenage birth rates (up to 2.64 pregnancies per 1000 females 10–14 years old) in Eastern Europe may be attributed to the difficulty of gaining access to contraception and the cultural restrictions that limit its usage [11]. According to previous research studies, rates of protected first sexual intercourse were very low (38%), and the condom was the main contraceptive method in this group (34%) [7]. In our study, barrier methods were significantly more often indicated by younger women as those of highest efficacy. Overestimation of the efficacy of condoms was also reported by other researchers [19].

Most previous studies focused on the rates of use or the trends in commonly used contraceptive methods. None of the published studies have compared women’s awareness of the physiology of the menstrual cycle with the use of natural contraceptive methods. Among sexually active Poles of early reproductive age the most frequently reported sources of information about contraception were the internet (64%) and magazines (54%), while professionals’ opinions were also mentioned by 53% of the respondents [19]. 8.9% of women who took part in our survey found natural contraceptive methods to be one of the most effective. 17.4% of respondents recommended them to their friends and acquaintances as sufficiently effective. Moreover, the efficacy of NFP was significantly more often emphasized by women who had never previously used any other form of contraception (20.1% vs 6.7%). Limited data supports the effectiveness of NFP, and most previous studies emphasized a significantly lower efficacy for NFP in comparison with hormonal contraceptives or intrauterine devices [20, 21]. The real efficacy of fertility awareness-based methods of contraception remains unknown [22]. the main disadvantages of NFP are that it requires the individual to give a lot of attention to their cycle, it is more vulnerable to disrupting factors, and it is time-consuming.

our results demonstrate that there is moderate awareness of infertility among women in the Polish population. 63.9% of women who took part in the study knew the definition of infertility. Sociodemographic factors, such as lower education levels, living in a smaller city and parity, significantly impacted the number of correct answers about this issue. Populations in the first to factor groups require extensive educational support on this topic. 80.8% of participants believed that in vitro fertilization is an effective method and should be reimbursed in Poland – thus indicating that there is a strong expectation for wider access to and reimbursement of infertility services in Poland.

According to the Polish Society of Gynecologists and Obstetricians a simple Pap smear carried out every 3 years, on every woman between the ages of 21 and 65, provides an acceptable level of screening for cervical cancer [23]. These recommendations are convergent with the statement of American College of Physicians [24]. 95.2% of the respondents in our study underwent a Pap smear screening test within the past 3 years and 81.9% thought it should be performed once a year. However, national registries of the rates of cervical cancer screening in Poland do not report such high levels of testing, therefore our data seems not to be representative of the Polish situation. Official figures indicate that attendance rates have been gradually increasing, from 24% in 2006 to 42% in 2015, but that they are still unsatisfactorily low, despite targeted recruitment methods (e.g., personal invitations) [25]. Apart from screening methods, primary prophylaxis is the best option for cervical cancer prevention. However, our study showed that only 3% of participants are aware of all the risk factors of cervical cancer that were indicated in our questionnaire. HPV infection was the best-known risk factor and it was indicated by 92.8% of the women. Previous studies have also demonstrated a high awareness of this particular risk factor because, in one of these studies, up to 76.4% of respondents declared to have heard about HPV [26]. Therefore, though Polish women appear to be quite familiar with the issue of cervical cancer prevention, better education concerning primary prophylaxis is recommended.

The authors agree that there are several limitations of our study. First, data in the paper are derived from self-reported questionnaires, which may give rise to a degree of bias. However, the anonymity of participation may have minimized the risk of women not being totally truthful in answer to some personal questions. Some other concerns arise from the nature of the study. Nonetheless, many outcomes and risk factors can be assessed at the same time. Cross-sectional studies are widely used to assess public health issues. The questionnaire was shared by using social media on the internet, therefore there are some concerns whether the results we obtained are representative of the entire population of Polish women. However, due to the now widespread access to and use of social media this might not be a significant limitation.

## Conclusions

Levels of knowledge about key aspects of reproductive health among Polish women of reproductive age seem quite satisfying. However, there is a need to improve comprehensive reproductive health education in Poland, especially among women at the lower educational levels and living in small centers. It seems crucial to focus on educational programs regarding reproductive issues that can be provided by medical professionals and implemented in schools in future.

## Additional file

Additional file 1: Questionnaire. An English version of the questionnaire used in the study. (DOCX 27 kb)

## Data Availability

The datasets used and/or analysed during the current study are available from the corresponding author (Iwona Szymusik, MD, PhD) upon reasonable request.

## Abbreviations

aOR: adjusted odds ratio
CI: Confidence interval
HPV: Human papilloma virus
IVF: In-vitro fertilization
K: thousand (e.g. 500 k = 500 thousand)
N: Number of participants
NFP: Natural family planning
Pap test: Papanicolaou test
SAS: Statistical Analysis System
SD: Standard deviation
STD: Sexually transmitted diseases
WHO: World Health Organization
y.o.: years old
