# Supplementary material for: Sex education in Poland – a cross-sectional study evaluating over twenty thousand polish women’s knowledge of reproductive health issues and contraceptive methods
Source: BMC Public Health. 2019 Jun 3;19:689. doi: 10.1186/s12889-019-7046-0 (PMC6547576; doi:10.1186/s12889-019-7046-0)
Supplement: Supplementary file 1 — Questionnaire. An English version of the questionnaire used in the study. (DOCX 27 kb) [file 12889_2019_7046_MOESM1_ESM.docx]

1. **What is the average length of a menstrual cycle?**
2. 20 +/- 5 days
3. 24 +/- 6 days
4. 28 +/- 7 days
5. 35 +/- 10 days
6. **What day is the first day of the menstrual cycle?**
7. The last day of menstrual bleeding.
8. The day when ovulation occurs.
9. The first day of menstrual bleeding.
10. It depends on the patient’s choice.
11. **On which day does ovulation occur?**
12. Immediately after the end of menstruation.
13. Usually 14 days before the next period.
14. About the 20th day of the cycle (if it is regular, 25 - 30 days).
15. 7 days before the expected menstruation
16. **How long does the average menstrual bleeding last and what is the average blood loss?**
17. About 3-5 days and 30-70 ml.
18. About 5-7 days and less than 30 ml.
19. About 7-10 days and less than 30 ml.
20. It doesn’t matter how long the menstruation lasts.
21. **In which phase is conception most likely to occur during unprotected intercourse?**
22. During the first (follicular) phase of the cycle.
23. At the time of ovulation.
24. During the second (luteal) phase of the menstrual cycle.
25. During menstrual phase.
26. **Which part of the genital tract is the most common site of fertilization?**
27. Vagina
28. Uterus
29. Fallopian tube
30. Ovary
31. **When does the basal body temperature increase during the menstrual cycle?**
32. During menstruation.
33. At the time of ovulation.
34. In the second phase of the menstrual cycle (after ovulation).
35. The cycle phase has no influence on basal body temperature.
36. **Have you ever used any contraceptive methods before?**

**□ Yes □ No**

1. **Which of the contraceptive methods listed below are characterized by the highest effectiveness?**
2. Natural contraceptive methods/ family planning (daily readings of the body temperature, observation of cervical mucus).
3. Oral hormonal contraception
4. Emergency (postcoital) contraception
5. Hormonal contraception as implants, patches.
6. Spermicides
7. Barrier contraception (condom / vaginal cap)
8. Intrauterine devices
9. **Do you think that natural contraceptive methods / family planning (daily readings of your body temperature, observation of cervical mucus) are sufficiently effective so that you would recommend them to your friends? □ Yes □ No**
10. **When you can expect a positive pregnancy test at the earliest?**
11. The day after fertilization
12. 3-5 days after unprotected sexual intercourse
13. 10- 14 days after fertilization
14. On the day corresponding to the next menstrual period
15. **Do you know the definition of infertility? □ Yes □ No**
16. **How long must the problem to conceive exist before one can diagnose infertility?**
17. 3 months
18. 6 months
19. 12 months
20. 24 months
21. It depends on the number of unprotected intercourses
22. **Do you think that in vitro fertilization is effective and should be reimbursed in Poland?**
23. It is effective and should be reimbursed.
24. It is effective but it should not be reimbursed.
25. It is not effective and should not be reimbursed.
26. It should be forbidden.
27. **How old are you? ………………………………………………………….**
28. **Are you pregnant / have you ever been pregnant?**

**□ Yes (**if yes, how many times ……………… ) **□ No**

1. **How often do you have gynecological appointments / consultations? (excluding pregnancy)**
2. Several times a year
3. Once a year
4. Every 2-3 years
5. Less frequently than every 2-3 years
6. **When was the last time you had Pap smear taken? …………………….months/years ago**
7. **How often should Pap smear be performed?**
8. Every six months
9. Every year
10. Every 3 years
11. Lack of recommendations, it depends on the woman's initiative
12. **Which of the following are the risk factors for cervical cancer? (you can mark more than one answer)**

- Early initiation of sex life
- Numerous sexual partners
- Pregnancy at a young age
- Smoking
- Papillomavirus infection (HPV)

1. **Please indicate your education level:**

**□ basic □ secondary □ vocational □ college / university**

1. **Place of residence:**
2. up to 10k inhabitants
3. 10k-100k inhabitants
4. 100k-500k inhabitants
5. cities > 500k inhabitants
